# Silicon improves root functioning and water management as well as alleviates oxidative stress in oilseed rape under drought conditions

**DOI:** 10.3389/fpls.2024.1359747

**Published:** 2024-02-21

**Authors:** Diana Saja-Garbarz, Marta Libik-Konieczny, Franciszek Janowiak

**Affiliations:** The Franciszek Górski Institute of Plant Physiology, Polish Academy of Sciences, Kraków, Poland

**Keywords:** *Brassica napus* var. *napus*, water content regulation, water deficit, aquaporins, abiotic stress

## Abstract

**Introduction:**

The aim of our study was to examine how silicon regulates water uptake by oilseed rape roots under drought conditions and which components of the antioxidant system take part in alleviating stress-induced ROS generation in the roots.

**Methods:**

The study analyzed mainly the changes in the roots and also some changes in the leaves of oilseed rape plants, including total silicon content, relative water content, osmotic potential, stomatal conductance, abscisic acid level, the accumulation of BnPIP1, BnPIP2-1-7 and BnTIP1 aquaporins, and the activity of antioxidant enzymes.

**Results and discussion:**

It was shown that plants growing in well-watered conditions and supplemented with silicon accumulate smaller amounts of this element in the roots and also have higher relative water content in the leaves compared to the control plants. It was demonstrated for the first time that BnTIP1 accumulation in oilseed rape roots is reduced under drought compared to wellwatered plants, and that this effect is intensified in plants supplemented with silicon. In addition, it was shown that silicon supplementation of oilseed rape increases catalase activity in the roots, which correlates with their high metabolic activity under drought and ultimately stimulates their growth. It was shown that silicon improves water balance in oilseed rape plants subjected to drought stress, and that an important role in these processes is played by tonoplast aquaporins. In addition, it was demonstrated that silicon reduces oxidative stress in roots under drought conditions by increasing the activity of catalase.

## 1 Introduction

Among abiotic stresses, drought stress is recognized as a major cause for low yielding of crops, causing 40-60% of total losses worldwide. In oilseed rape, the stress associated with water scarcity is most harmful in the generative phase of plant growth, but its effects are also visible earlier in the course of vegetative development. Drought causes the following physiological changes in oilseed rape: an increase in the level of abscisic acid (ABA), stomata closure, a decrease in CO_2_ diffusion and assimilation, an increase in the level of reactive oxygen species (ROS), as well as a decrease in chlorophyll content and enzymatic activity, which translates into disturbances in plant growth and deterioration of yielding components (Raza et al., 2017). For over a decade much promise for facilitating plant adaptation to the conditions of soil drought has been attached to silicon (Si) supplementation. Numerous studies have highlighted the beneficial role of silicon in various aspects, including its capacity to mitigate stresses imposed by heavy metal exposure. This involves mechanisms such as the reduction of metal ions in the soil substrate, co-precipitation of toxic metals, regulation of metal-transport-related genes, chelation, stimulation of antioxidants, compartmentation of metal ions, and structural alterations in plants (Bhat et al., 2019; Coskun et al., 2019; Zargar et al., 2019). Understanding these processes provides valuable insights into optimizing silicon-related interventions for diverse plant species. A crucial aspect of research involves refining silicon-based interventions, optimizing application methods, and integrating silicon supplementation with other agricultural practices. This is particularly pertinent in the case of agriculturally significant plants. Silicon supplementation in sugarcane has been demonstrated to confer resistance against various stresses, including water stress, cold, arthropod invasion, and fungal infections (Misra et al., 2023). This highlights the potential for silicon to play a vital role in enhancing the resilience of crops, offering valuable implications for sustainable agricultural practices. It has been shown that under drought conditions silicon improves water uptake and plant hydraulic conductivity as well as increases polyamine accumulation while decreasing ethylene production in sorghum (Sonobe et al., 2009), in meadow grass (*Poa* L.) it deposits in the leaves, helping them maintain a vertical position, and thus increasing their photosynthetic efficiency (Saud et al., 2014), in cucumber it helps to maintain the rate of photosynthesis, reduces stomatal conductance, improves water holding capacity, stabilizes transpiration and reduces chlorophyll degradation (Ma, 2004), in wheat it increases the activity of catalase (CAT), superoxide dismutase (SOD) and glutathione reductase (GR) (Gong et al., 2005), in sunflower it increases relative water content (RWC) (Gunes et al., 2008), and finally, in many species it increases the accumulation of proline, glycine and betaine (Ahmad and Haddad, 2011), and depending on the species, it deposits in the cell walls and also reduces transpiration (Mandlik et al., 2020). According to the latest reports, silicon deposits in plants by creating a mechanical barrier (Coskun et al., 2019), but also in the form of various silica cells, e.g. phytolites (Kumar et al., 2020). Its amount strongly depends on the type of tissue, with the greatest accumulation observed in the cell wall (Mandlik et al., 2020). In the roots, as it was for the first described in rice by Parry and Soni (1972), silicon is deposited in the endoderm, though it is not the only location (Bokor et al., 2019). Studies on rice have also shown that very small amounts of silicon can alter the structure of the cell wall (He et al., 2015). Isa et al. (2010) showed that the deposition of Si may result in the formation of complexes of this element with polysaccharides (including hemicelluloses) in the cell wall. After being initially absorbed by the roots, silicon is transported to the shoots through the xylem (Mandlik et al., 2020).

Soil water deficit affects the roots capacity for water uptake, which may in many cases be more important than the regulation of water loss in the leaves (Aroca et al., 2012). Water absorption by the roots is largely dependent on root conductivity (Steudle, 2000), which has been demonstrated, among others, in sorghum (Liu et al., 2014), rye (Hattori et al., 2009), tomato (Shi et al., 2016), and cucumber (Zhu et al., 2015) under various abiotic stresses. In many species, silicon stimulates the osmotic driving force through the accumulation of soluble sugars and amino acids (Sonobe et al., 2010), which has been demonstrated, among others, in rice (Ming et al., 2012) and oilseed rape (Habibi, 2014) under drought conditions, although this tendency is not observed in all plant species (Liu et al., 2015; Shi et al., 2016). In addition to the above-described mechanisms in the regulation of water permeability through the roots, a key role in the impact of stress on the plant’s water balance is also played by Aquaporins (AQP) (Maurel et al., 2008). Of great importance in this respect are Plasma Membrane Intrinsic Proteins (PIPs) as well as Tonoplast Intrinsic Proteins (TIPs) (Kapilan et al., 2018). The PIPs play a crucial role in regulating the uptake and loss of water by cells. Among the PIPs, PIP1 aquaporins are generally considered less efficient as water channels compared to PIP2 aquaporins. The PIP2 subgroup, on the other hand, is believed to provide effective water communication between cells. In addition to PIPs, TIPs situated around the tonoplast, contribute to the maintenance of constant cell turgor pressure and the regulation of the osmotic balance of the cells (Kapilan et al., 2018). Notably, during abiotic stress conditions, certain AQP’s isoforms can be activated in specific plant tissues or throughout the entire plant. The activation of these AQPs plays a role in regulating the plant’s response to the imposed stress. The changes in the expression of their respective genes are particularly significant in the plant’s water transport. In sorghum it was observed that silicon increases the expression of PIP genes, thus increasing water uptake under drought (Liu et al., 2014). This adaptive response involving AQP is a crucial mechanism by which plants modulate water movement and osmotic balance to cope with environmental challenges.

Based on the above-mentioned results, Chen et al. (2018) proposed possible mechanisms of water balance regulation in response to plant supplementation with silicon. It is presumed that Si improves aquaporin activity by up-regulating the expression of *PIP* genes and alleviating ROS-induced inhibition of aquaporin activity. Moreover, it may also increase the accumulation of soluble sugars and/or amino acids in the xylem sap through osmoregulation. The osmolyte accumulation in the xylem sap increases the osmotic driving force. Consequently, silicon may adjust root growth and increases the root/shoot ratio, which together with the enhancement of aquaporin activity and osmotic driving force contribute to the improvement of root hydraulic conductance. Higher root hydraulic conductance results in increased uptake and transport of water, which in turn improves plant resistance to water deficiency (Chen et al., 2018). The issue addressed in this study is all the more important because according to specialists’ predictions, the coming decades will bring unavoidable alterations in the interactive effects of abiotic and biotic stresses on plants due to global climate changes (Surówka et al., 2020). The results derived from research conducted on the above-ground parts of oilseed rape plants reveal that, despite being regarded as a weak silicon accumulator, silicon supplementation holds substantial significance (Saja-Garbarz et al., 2021, 2022). This supplementation exerts a positive influence on the regulation of water management and aids in alleviating the effects of oxidative stress in oilseed rape plants. Therefore, based on this studies we formulated and tested a hypothesis that in oilseed rape under drought stress silicon regulates water uptake, affecting root growth and alleviating ROS generation in the roots. The validation of this hypothesis contributes to a deeper understanding of the mechanisms underlying the response of plants to drought stress and sheds light on the protective role of silicon in mitigating the impact of such stress. Furthermore, the findings from our research hold potential utility in agriculture. By uncovering the positive effects of silicon supplementation in weak silicon accumulators, this research may contribute to the development of agricultural solutions aimed at improving crop yield and resilience, especially under conditions of water scarcity and environmental stress.

## 2 Materials and methods

### 2.1 Plant material, experimental design, and sampling

**Figure 1:**
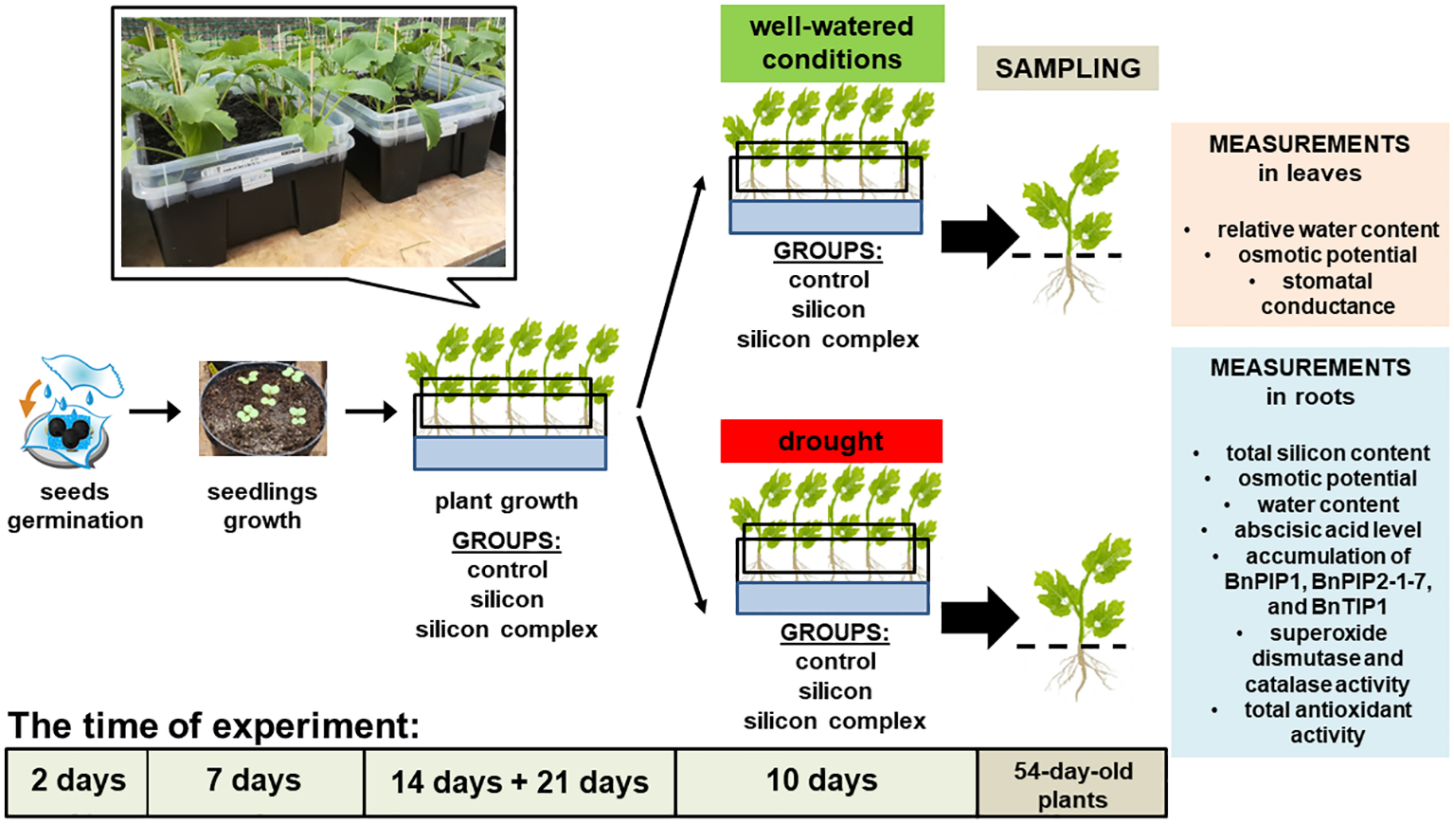
Experimental design.

Seeds of oilseed rape cv. Markus (obtained from the Institute of Plant Protection – National Research Institute, Poznań, Poland) were germinated according to the procedure described by (Saja-Garbarz et al., 2022). Markus is one of the most popular spring cultivar in Poland, recommended by COBORU – Research Centre for Cultivar Testing. Next, two-day-old seedlings were planted into pots (five into each pot), containing a mixture of soil and sand (garden soil/*chernozem*/sand; 1:2:1; v/v), with pH ca. 7.0 – 7.3, and grew in a growth chamber (25 ± 2°C, 14h photoperiod) for one week. 9-day-old plants were placed in black plastic boxes (10 in each box), 11.0** l** in volume, containing the same soil mixture as the pots. The boxes had numerous holes in the bottom to allow plant roots to grow out of them. Each box was placed in a second larger box with a solid bottom filled with Hoagland solution (1:1). The double box system made the roots grow in the soil mixture contained in the inner box, through the holes in its bottom, and into the Hoagland solution in the outer box, creating a vertically split root system (**Figure 1**). The double box system not only allowed for better access to the roots required for analysis but also provided a means to replicate the most natural growth conditions for plants.

The plants grew in a growth tunnel for 2 weeks (air temperature 25/16 ± 4°C day**/**night, relative air humidity 37 – 80%) and were watered. 23-day-old plants with two leaves unfolded [growth stage: leaf development, code 12 on the BBCH-scale (canola) (Meier, 2001)] were divided into three groups – control (plants watered with plane water), silicon group (plants watered with orthosilicic acid tetraethyl ester; Sigma-Aldrich, St. Louis, Missouri, USA) and silicon complex group (plants watered with *Optysil* preparation; Intermag, Olkusz, Poland) (Saja-Garbarz et al., 2021, 2022). *Optysil* is a commercially available plant growth stimulator, also contains iron (in ethylenediaminetetraacetic acid, EDTA chelate form) in the concentration of 0.00027 mM FE at the second oxidation stage but does not contain any other ions. The silicon concentration in both solutions was the same at 3.4 mM Si. Over the next three weeks of growth the plants were watered with appropriate solutions (aqueous silicon solution in the appropriate concentration for each type of silicon) three times at weekly intervals, 440 ml per box. As previously mentioned, Hoagland’s nutrient solution (1:1) was added to each outer box in such an amount that the roots which had grown through the bottom of the inner box were immersed in the solution. The solution was replenished every day.

44-day-old plants were further split into two subgroups – the first continued growing in well-watered conditions and the second was subjected to drought over the course of 10 days. In the second subgroup the drought effect was initiated by adding 44,58 gL^-1^ mannitol solution to the Hoagland’s medium (Michel et al., 1983; Neto et al., 2004). To simulate drought stress similar to soil drought at a level of 30-40% of the full field capacity of the specified soil, as used in prior studies (Saja-Garbarz et al., 2021, 2022), an osmotic potential ranging approximately from -0.6 to -0.7 MPa was targeted. For this purpose, 44.58 g of mannitol was dissolved in 1 liter of distilled water. Subsequently, the osmotic potential was measured to verify that the values of this parameter were accurate. The mannitol solution was replaced every 2 days (5 times in total). Leaves and roots of 54-day-old plants were sampled for analysis. We investigated the effect of silicon on the total silicon content, relative water content (RWC), osmotic potential, stomatal conductance, abscisic acid level, accumulation of BnPIP1, BnPIP2-1-7 and BnTIP1 aquaporins, the activity of antioxidant enzymes – superoxide dismutase (SOD) and catalase (CAT), as well as total non-enzymatic antioxidant activity in leaves and roots or only in roots of oilseed rape plants.

### 2.2 Relative water content in leaves

An individual sample consisted of a leaf (sixth from the bottom) cut into four pieces. The freshly cut leaves were weighed (FW) and then immersed in 15 ml of distilled water in 20 ml screw-cap scintillation vials. Next the samples were shaken for 24 h at room temperature. Afterwards, they were weighed to determine the full turgor weight (TW) and then dried at 70°C for 72 h to determine the dry weight (DW). The relative water content (RWC) was calculated according to the formula (Turner, 1981): RWC = (FW-DW)/(TW-DW) x 100. For each treatment two independent pool samples were collected (two biological replicates) and five measurements were performed on each of them (five technical replicates), giving in total ten measurements per treatment.

### 2.3 Osmotic potential in leaves and roots

Osmotic potential (Ψo) was measured using the dew point method and the HR 33T microvoltmeter (Wescor, Logan, UT, USA) as described by Saja-Garbarz et al. (2021). Osmotic potential measurements were performed on filter discs moistened with cell sap. Both measurements were performed on the second fully developed leaf from the top. For each treatment three independent pool samples were collected (three biological replicates) and three measurements were performed on each of them (three technical replicates), giving in total nine measurements per treatment.

### 2.4 Leaf stomatal conductance

The stomatal aperture status of leaves was assessed on the measurements with a diffusion porometer on the abaxial leaf surface and expressed as leaf stomatal conductance (mmol m^−2^ s^−1^). Leaf stomatal conductance was measured approximately 4 h after the start of the photoperiod using a handheld AP4 porometer (Delta-T Devices, Cambridge, UK) as described by Saja-Garbarz et al. (2021). For each treatment nine measurements were performed, each on the fifth oldest leaf of a different plant.

### 2.5 Total silicon content in roots

Si content analyses were carried out according to the ICP-OES method described by Saja-Garbarz et al. (2021). The roots were cut and burnt in a open mineralizer in borosilicate glass. For each treatment three independent pool samples of roots (whole roots from all zones) were collected from ten different plants (three biological replicates) and three measurements were performed on each of them (three technical replicates), giving in total nine measurements per treatment.

### 2.6 Abscisic acid level in roots

Three pool root samples (whole roots from all zones) were collected per treatment, each coming from at least three different plants, dried using paper tissues, weighed (FW) and then frozen. Next, they were freeze-dried and ground with ball mill MM400 (Retsch, Haan, Germany) in Eppendorf vials. After adding 1.5 mL of cold distilled water, the vials were heated for 3 min in a thermoblock set to 90°C and then shaken overnight at 4°C in order to extract ABA (Quarrie et al., 1988). The next day, after the centrifugation of the aqueous extracts for 20 min in a refrigerated centrifuge at 18,000 × g (MPW-350R, Warsaw, Poland), ABA was measured in the supernatant by means of indirect enzyme-linked immunosorbent assay (ELISA) according to Walker-Simmons and Abrams (1991), using MAC 252 antibody (Babraham Technix, Cambridge, UK). Absorbance was determined with microplate reader Model 680 (Bio-Rad Laboratories, Hercules, CA, USA) at the wavelength of 405 nm. Three ELISA measurements were performed for each sample giving in total nine ABA measurements for each treatment.

### 2.7 Protein concentration in crude root extract

The roots (whole roots with all zones) of oilseed rape plants were cut into fragments, and the samples prepared according to the method described by Saja-Garbarz et al. (2022). Samples were homogenized in buffer (2.5 mL of a Tricine buffer containing 100 mM Tricine, 3 mM MgSO4, 1mM DTT, 3 mM EGTA, adjusted to pH 8.0 with 1 M Tris) at 4 °C, centrifuged and the supernatant was used for the analyses. Protein concentration in the obtained crude extract was measured according to Bradford (1976). For each treatment three independent pool samples (pool sample comprises roots from 9-10 different plant for each treatment) were collected (three biological replicates) and two measurements were performed on each of them (two technical replicates), giving in total six measurements per treatment.

### 2.8 Accumulation of BnPIP1, BnPIP2-1-7, and BnTIP1 aquaporins in roots

The measurement of aquaporin accumulation was performed on 10 µg of total proteins extracted from roots. Electrophoretic separation, protein transfer into a nitrocellulose membrane, membrane blocking, incubation in the primary antibody (anti-PIP1, anti-PIP2-1-7, anti-TIP1; Agrisera) and in the secondary conjugated anti-rabbit antibody (Sigma-Aldrich) and also visualization of protein bands corresponding to PIP1, PIP2-1-7 and TIP1 were performed using methods developed for leaves by Saja-Garbarz et al. (2022). Blots were scanned with the Epson Perfection V700 Photo scanner (Epson America, Long Beach, CA, USA). Staining intensity of the bands corresponding to PIP1, PIP2-1-7 and TIP1 was determined through densitometric analyses using ImageJ software (National Institutes of Health, Bethesda, Maryland, USA). For each treatment three independent pool samples were collected (three biological replicates) and one measurement was performed on each of them (one technical replicate), giving in total three measurements per treatment.

### 2.9 Superoxide dismutase and catalase activity in roots

The root material (total protein extract) was prepared in the same way as in the case of the analysis of aquaporins. The activity of SOD and CAT was visualized on 12% (SOD) or 10% (CAT) polyacrylamide gels. For native electrophoretic separation of protein fractions by the Laemmli (1970) method, a buffer system without sodium dodecyl sulfate (SDS) at 4°C and 180 V was used. 10 µg of total protein extract were used for the SOD analysis and 10 µg for the CAT analysis. The bands corresponding to SOD activity were visualized using the activity staining procedure described by Beaucham and Fridovic (1971) and in the case of CAT – using the method described by Woodbury et al. (1971). A detailed description of the methodology is presented in Saja-Garbarz et al. (2022). Gels were scanned with the Epson Perfection V700 Photo scanner (Epson America, Long Beach, CA, USA). Intensity of the bands corresponding to SOD and CAT activity was determined through densitometric analyses using ImageJ software (National Institutes of Health, Bethesda, Maryland, USA). For each treatment three independent pool samples were collected (three biological replicates) and one measurement was performed on each of them (one technical replicates), giving in total three measurements per treatment.

### 2.10 Total antioxidant activity in roots

Three pool root samples (whole roots from all zones) were collected per treatment, each coming from at least three different plants, dried using paper tissues, weighed (FW) and then frozen. Next, they were freeze-dried and ground with ball mill MM400 (Retsch, Haan, Germany) in Eppendorf vials. After adding 1 mL of 50% ethanol, the vials were shaken for 2 h at room temperature. After the centrifugation of the extracts for 20 min in a refrigerated centrifuge at 18,000 × g (MPW-350R, Warsaw, Poland), total antioxidant activity (free radical scavenging activity) was measured in the supernatant using 0.5 mM solution of stable free radical 1,1-diphenyl-2-picrylhydrazyl (DPPH, Merck, Darmstadt, Germany) in methanol according to the method by Brand-Williams et al. (1995) with some modifications adapting the protocol to 96-well microtitre plates (Żur et al., 2021). The results were expressed as µmoles of Trolox equivalents g^−1^ DW. Three DPPH measurements were performed for each sample giving in total nine measurements for each treatment.

### 2.11 Statistical analysis

Statistical analysis and graphic presentation of the results were performed using Statistica 13.1 (StatSoft, Tulsa, OK, USA). The main effects of the treatments on the physiological parameters were determined with one or two-factor analysis of variance (ANOVA). The significance of differences among the treatment means was calculated with Duncan’s multiple range test at 0.05 probability level. The significance of Pearson’s correlation coefficients (r)between the measured parameters was tested at p ≤ 0.1, p ≤ 0.05 and p ≤ 0.01. The figures include mean values ± standard deviation (SD). All statistical analyses for each parameter and the correlation between them are presented in the form of tables, which have been included in the **Supplementary Materials**.

## 3 Results

### 3.1 Silicon content and the effect of silicon supplementation on water management parameters of leaves and roots

The analyses of silicon content revealed a great diversity in the ability of oilseed rape roots to accumulate this element. Plants growing in well-watered conditions which were watered with water (control) had a significantly higher level of Si than those treated with silicon (**Table 1**).

**Table 1:** Silicon content and selected parameters of water management in oilseed rape plants.

Growth conditions	Treatment	Root silicon content (mg/kg)	RWC (%)	Leaf osmotic potential Ψo (MPa)	Stomatal conductance (mmol m^-2^ s^-1^)	Root osmotic potential Ψo (MPa)	Root abscisic acid level (nmol g^-1^ DW)
**well-watered conditions**	**control**	2271.50^a^	91.00^d^	-0.98^a^	241.71^a^	-0.23^b^	1.30^ab^
	**silicon**	541.50^c^	95.11^b^	-1.04^a^	197.49^a^	-0.16^a^	1.16^ab^
	**silicon complex**	745.20^b^	97.32^a^	-1.03^a^	181.50^a^	-0.17^a^	0.89^b^
**drought**	**control**	1086.16^b^	83.50^e^	-1.72^d^	136.27^b^	-0.31^d^	0.92^b^
	**silicon**	232.20^d^	93.04^c^	-1.34^b^	141.79^ab^	-0.29^d^	1.08^ab^
	**silicon complex**	2186.07^a^	90.00^d^	-1.41^c^	140.85^b^	-0.26^c^	1.46^a^

The impact of silicon supplementation was examined in roots and leaves of plants growing under well-watered and drought conditions. Control – plants not supplemented with silicon. The means (n = 9 for silicon content; n = 10 for leaf relative water content, n = 9 for leaf osmotic potential, n = 9 for stomatal conductance, n = 9 for root osmotic potential, n = 9 for root abscisic acid level) marked with the same letter do not differ significantly according to Duncan’s multiple range test, p < 0.05, applied collectively to all points of measurement.

Under drought, on the other hand, the highest Si content was found in the roots of plants supplemented with the *Optysil* preparation (silicon complex). The lowest silicon content in the roots was observed in plants watered with silicon alone in drought conditions (**Table 1**). In general, a reduction in silicon content was noted in the roots of plants subjected to drought conditions compared to optimal conditions, both in control plants and in those supplemented with orthosilicic acid. This phenomenon could be attributed to the increased transport of silicon upward in the plant under water deficit conditions, driven by its protective role in limiting transpiration, for instance, by incorporating into plant cell walls and reinforcing them. In plants supplemented with the silicon complex, the observed decrease might be influenced by the increased complexity of this process due to the presence of additional iron ions, which could potentially regulate the process differently.

The results of the assessment of water relations in the above-ground plant parts, based on measurements in leaves, are presented in **Table 1**. Relative water content (RWC) both in well-watered and drought conditions was significantly lower in plants untreated with silicon than in plants supplemented with this element (silicon and silicon complex) and the lowest in control plants under drought conditions. In well-watered conditions, no change in leaf osmotic (Ψo) and water (Ψw) potential was observed. Under drought the values of both potentials were lower than in well-watered conditions. The lowest values for both were observed in control plants under drought conditions. In well-watered conditions, stomatal conductance of control plants was significantly higher compared to plants supplemented with silicon. Under drought, the values of this parameter decreased significantly. Differences in root osmotic potential were associated with environmental conditions and with silicon supplementation, but in this case only in drought conditions. The roots of plants growing in well-watered conditions had a significantly higher osmotic potential than those growing under drought. It was also higher in plants which were supplemented with Si. Under drought conditions root osmotic potential was the highest in plants treated with silicon complex. The analysis of abscisic acid (ABA) content showed a tendency lower ABA level in the roots of control plants under drought compared to well-watered conditions. The differences in ABA content were not observed in plants supplemented with silicon alone. In the case of plants treated with silicon complex, the ABA level was lower in plants growing in well-watered conditions than in drought (**Table 1**). On the other hand, a noticeable increase in root growth resulting from Si treatment under drought compared to control plants (**Supplementary Figure 1**) was found indicating a significant difference in root metabolic activity.

### 3.2 Effect of silicon supplementation on the accumulation of BnPIP1, BnPIP2-1-7 and BnTIP1;1 aquaporins in the roots of oilseed rape plants growing in well-watered and drought conditions

**Figure 2:**
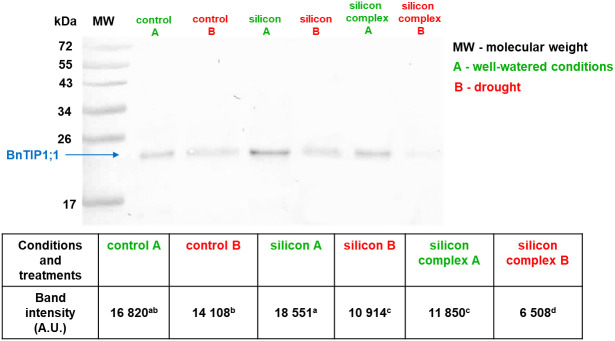
The impact of drought on the accumulation of BnTIP1;1 protein in roots of control and silicon-supplemented plants of oilseed rape. The band intensity corresponds with the level of protein identified with anti-TIP1;1 antibody. MW – molecular weight standard (Thermo Scientific PageRuler Prestained Protein Ladder), 10 µg of total proteins were applied on each lane. BnTIP1;1 accumulation was determined based on a densitometric analysis of the band intensity staining (table). The values are presented as arbitrary units (A.U.) which correspond to the area under the densitometric curves. Mean values (n=3) ± SD which do not differ significantly are marked with the same letter (Duncan’s multiple range test, p<0.05).

Changes in biochemical parameters of roots associated with water relations were investigated through an analysis of aquaporin accumulation (**Supplementary Figure 2**; **Figure 2**). In the case of PIP proteins, accumulation of both BnPIP1 (**Supplementary Figure 2A**) and BnPIP2-1-7 (**Supplementary Figure 2B**) was observed – see **Supplementary Material**. However, this identification was not sufficient to perform a densitometric analysis to determine the potential differences in their accumulation resulting from silicon supplementation. Moreover, in the case of BnPIP2-1-7, the presence of only one of the two possible dimers of this protein was ascertained (**Supplementary Figure 2B**) – see **Supplementary Material**.

Measurements of the accumulation of tonoplast aquaporin BnTIP1;1 are shown in **Figure 2**.

As in the case of BnPIP2-1-7, BnTIP1;1 was identified in the roots as a monomer. A comparison of BnTIP1;1 accumulation in the roots of plants growing in well-watered and drought conditions was performed as well as an analysis of changes induced by silicon supplementation. Under well-watered conditions, the densitometric analysis showed the highest level of BnTIP1;1 accumulation in the roots treated with silicon alone (**Figure 2**). On the other hand, a significantly lower level of its accumulation was observed in the roots of plants treated with *Optysil* (silicon complex). In contrast, under drought, a significantly lower BnTIP1;1 content was detected in all treatments compared to well-watered conditions. Moreover, under drought, plants supplemented with Si, regardless of whether it was silicon or silicon complex, had a significantly lower level of BnTIP1;1 accumulation compared to control plants. Similar to well-watered conditions, the lowest value was noted in the case of plants supplemented with the silicon complex (**Figure 2**). The significantly reduced accumulation of TIP proteins in drought compared to optimal conditions, especially after silicon supplementation, indicates their active participation in the regulation of the osmotic balance in the cells, and thus proves that, despite the fact that oilseed rape is classified as a weak silicon accumulator, the influence of this element on water management parameters is significant in this plant. Under drought stress, the down-regulation of the TIP genes has been described in several studies (Kurowska et al., 2019). This expression pattern lead to a decrease in TIP accumulation and further diminishes the water permeability of the tonoplast in order to avoid water loss and to minimise the water flow through the cell membranes.

In summary, the examination of PIP aquaporin proteins in oilseed rape roots does not yield a conclusive answer regarding changes in their accumulation due to silicon supplementation, both under well-irrigated conditions and during drought. However, in the case of TIP aquaporins, significant differences in protein content are particularly noticeable depending on the growth conditions, with a notable reduction in accumulation during drought. This effect is more pronounced in plants supplemented with silicon.

### 3.3 Effect of silicon supplementation on the activity of superoxide dismutase and catalase in the roots of oilseed rape plants growing in well-watered and drought conditions

**Figure 3:**
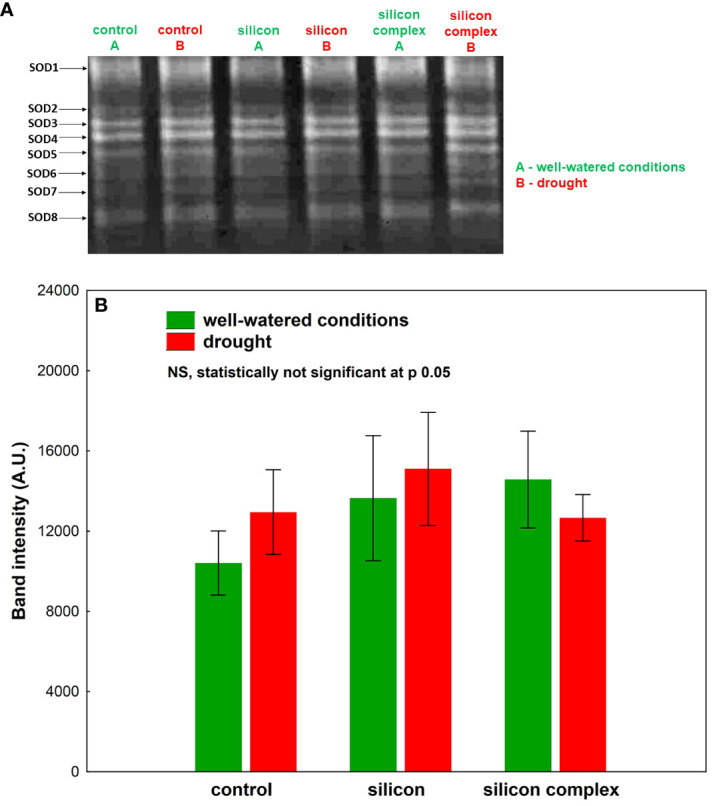
The impact of silicon supplementation on SOD activity in protein fraction isolated from the roots of oilseed rape plants growing in well-watered and drought conditions. Control – plants not supplemented with silicon. The band intensity corresponds with the level of protein identified with the activity of SOD isoforms on the basis of selective inhibitory staining; 10 µg of total proteins were applied on each lane **(A)**. The activity of SOD was determined based on a densitometric analysis of the total band intensity corresponding with different SOD isoforms **(B)**. The values are presented as arbitrary units (A.U.) which correspond to the area under the densitometric curves. Mean values (n=3) ± SD which do not differ significantly are marked with the same letter (Duncan’s multiple range test, p<0.05).

An analysis of the activity of superoxide dismutase (SOD) isolated from oilseed rape roots showed no significant differences among the investigated treatments (**Figure 3**). In-gel Visualisation of superoxide dismutase (SOD) isolated from oilseed rape roots revealed presence of several isoforms of this enzyme. The total superoxide dismutase activity did not differ significantly among the investigated samples.

**Figure 4:**
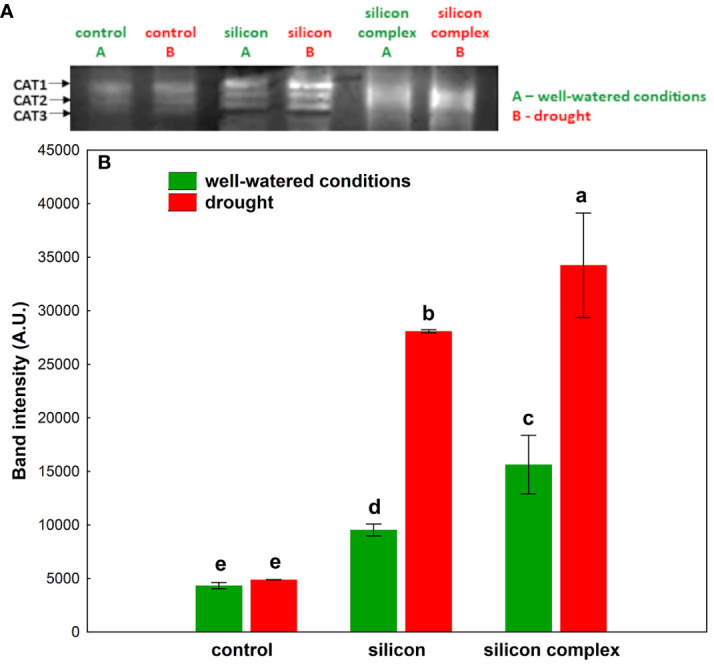
The impact of silicon supplementation on CAT activity in protein fraction isolated from roots of oilseed rape plants growing in well-watered and drought conditions. Control – plants not supplemented with silicon. The band intensity corresponds with CAT activity; 10 µg of total proteins were applied on each lane **(A)**. The activity of CAT was determined based on a densitometric analysis of the band intensity **(B)**. The values are presented as arbitrary units (A.U.) which correspond to the area under the densitometric curves. Mean values (n=3) ± SD which do not differ significantly are marked with the same letter (Duncan’s multiple range test, p<0.05).

A densitometric analysis of band intensity confirmed that the activity of SOD was similar in well-watered and drought conditions (**Figure 3B**). Moreover, no changes resulting from Si supplementation were observed. A measurement of catalase (CAT) activity performed on a polyacrylamide gel showed a higher activity of this enzyme in the roots of silicon-treated plants compared to the control (**Figure 4A**).

A densitometric analysis showed that in well-watered conditions the activity of CAT was two-fold higher in plants supplemented with Si and three-fold higher in plants supplemented with the silicon complex compared to the control (**Figure 4B**). Under drought conditions no significant changes in CAT activity were observed in the roots of control plants compared to well-watered conditions, but there was a significant increase in the activity of this enzyme in silicon-treated plants.

### 3.4 Effect of silicon supplementation on total non-enzymatic antioxidant activity in the roots of oilseed rape plants growing in well-watered and drought conditions

**Figure 5:**
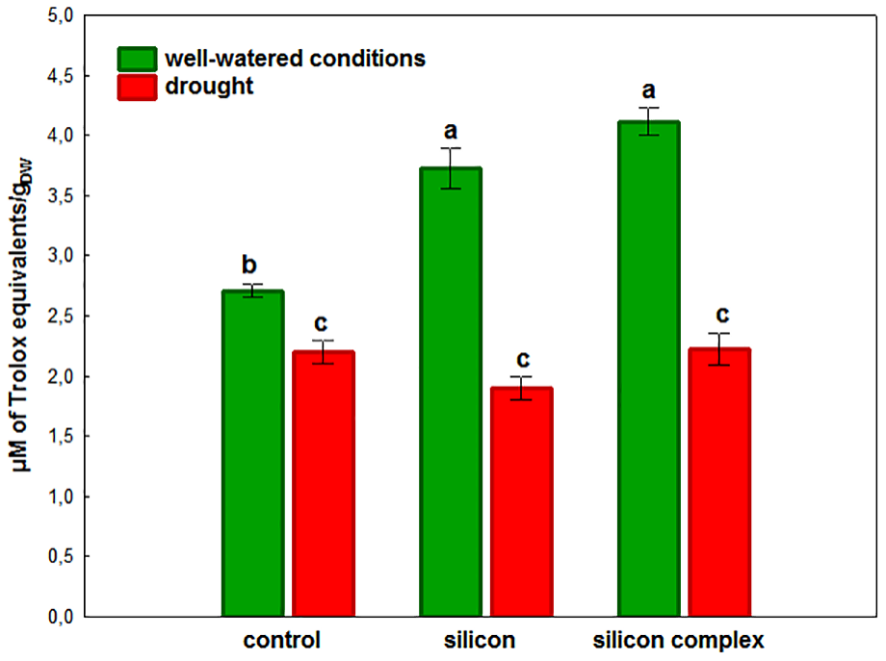
The impact of silicon supplementation on total non-enzymatic antioxidant activity in roots of oilseed rape plants growing in well-watered and drought conditions. Control – plants not supplemented with silicon. Mean values (n=9) ± SD which do not differ significantly are marked with the same letter (Duncan’s multiple range test, p<0.05).

A complementary evaluation of the antioxidant activity in oilseed rape roots was performed by analyzing the activity of low molecular weight non-enzymatic antioxidants (**Figure 5**).

It was shown that in well-watered conditions Si supplementation significantly increases the activity of these antioxidants in the roots of plants treated with silicon and silicon complex, in the second case the value was the highest. In contrast, under drought there was a significant decrease in low molecular weight antioxidant activity in all treatments compared to well-watered conditions. Moreover, the activity of these non-enzymatic antioxidants was comparable in the roots of control plants and those supplemented with silicon.

Under well-watered conditions Si and Si complex treatments reduced ABA level in the roots compared to the control, which indicates changes in the osmotic potential in the leaves of oilseed rape plants under different growth conditions correlated positively and significantly with other water relations parameters i.e. root osmotic potential (Tab_SM6) as well as with the level of BnTIP1;1 aquaporins and negatively with the activity of CAT. Interestingly, stomatal conductance correlated significantly and negatively with the activity of SOD and CAT.

## 4 Discussion

Water scarcity is one of the main environmental factors limiting plant growth (Raza et al., 2017; Chen et al., 2018; Mandlik et al., 2020). Many years of research based on the supplementation of plants with silicon have proven that it protects plants against the adverse effects of drought, improving their hydration and water use efficiency (Zhu et al., 2015). Certainly, the diverse array of responses observed in plants to silicon supplementation primarily stems from variations in their ability to absorb and accumulate this element. The uptake and transportability of silicon are primarily influenced by the plant’s roots and the chemical composition of the soil (Mandlik et al., 2020). This process is not only dependent on the plant species but also on environmental conditions and the specific phase of plant development. Undoubtedly, the molecular mechanism of silicon uptake plays a crucial role in shaping plant responses to silicon supplementation. Studies conducted thus far have revealed significant variability in the silicon transporters discovered among different plant species and even within tissues of the same plant. This variability directly impacts the potential efficacy of harnessing the protective role of silicon by the plant. Understanding these molecular mechanisms is essential for optimizing silicon supplementation strategies and enhancing its beneficial effects on plant growth and stress response. Although oilseed rape is categorized as a weak Si accumulator (Mandlik et al., 2020), silicon plays an important role in protecting the plants of this species against drought stress (Saja-Garbarz et al., 2021, 2022). Oilseed rape plants supplemented with silicon accumulate it in the above-ground part to a much greater extent than control plants, both under well-watered conditions and periodic soil water shortage (Saja-Garbarz et al., 2021). So far, it has not been described in what form silicon is accumulated in rapeseed, while in species that are strong accumulators of this element, it is accumulated in the roots, in cell walls (Sonah et al., 2017). The presented research revealed that plants growing in well-watered conditions and supplemented with silicon accumulate it in the roots to a smaller extent than control plants (**Table 1**). Such an effect may be explained by increased transport of this element upwards to the plant shoot. Under well-watered conditions, Si level in the roots seems to be dependent more on stomatal conductance (transpiration rate) than on the concentration of this element in the soil or medium – the highest Si level was observed in the control, which had the highest stomatal conductance. Under drought with stomata partially closed, Si level in the roots seems to be regulated by other unknown factors which need further investigation. Si uptake by the roots occurs by means of appropriate transporters, e.g. Lsi 1 and Lsi 2. It is then directed to the xylem, from where it is uploaded and transported to various parts of the plant by other transporters, e.g. Lsi 6 (Mandlik et al., 2020). In plants growing under well-watered conditions, this process occurs without disturbances. Under drought, however, due to disturbances in transport, the differences in silicon accumulation are much more noticeable (**Table 1**), which may be associated with the plant’s adjustment of its water-saving strategy. This hypothesis seems probable as the analysis of water management parameters in the above-ground plant part showed typical mechanisms associated with the plant’s growth in specific environmental conditions. Thus, plants supplemented with silicon growing in well-watered conditions had higher relative water content (RWC) in the leaves compared to control plants, though no changes in the leaves’ osmotic potential was observed (**Table 1**). These results also correlate with the previously published results of the characterization of aerial parts of plants in the work of Saja-Garbarz et al. (2021), which, due to the different experimental system used, were repeated to characterize the condition of water management and the response to drought stress stimulated by mannitol. On the other hand, Si supplementation did not cause any significant changes in oilseed rape roots. Moreover, it was observed that soil water shortage caused a decrease in the osmotic potential, which is a known strategy of plant adaptation to stress associated with water deficit in the environment (Hsiao et al., 1976), though the unambiguousness of this correlation differs between species. As early as in 1977 Morgan et al. demonstrated that plant species differ in their innate basic capability for osmotic regulation, in particular proving that this variability may even apply to different genotypes of one crop species. Although osmotic adjustment is usually measured in the shoot, it is also observed in the root. Under well-watered conditions Si and Si complex treatments tendence reduced ABA level in the roots compared to the control, which could indicate intensive ABA transport to the above-ground plant part causing partial stomata closure (**Table 1**). On the other hand, under drought conditions, these treatments tend to increase the ABA level in the roots, while stomatal conductance does not change compared to the control. Additionally, it was demonstrated that under drought conditions, silicon supplementation increased the root size of oilseed rape plants (**Supplementary Figure 1**), enhancing their capacity for water and mineral uptake from the soil. This finding is consistent with results reported in previous studies, such as those conducted in sorghum (Hattori et al., 2005, 2009; Ahmed et al., 2011), rice (Ming et al., 2012) and soybean (Abdullah et al., 2023). The ability to maintain root growth as soil water content declines is reported in various types of crop roots. This is an important characteristic of plant roots when resources are insufficient under drought conditions. Different cellular metabolism mechanisms such as osmotic regulation, cell-wall modification, and antioxidative system activity have to be involved in such responses. Osmotic adjustment maintains cellular water balance and homeostasis in root tissues, which reduces the damage caused by water losses and maintains the turgor of root cells. Simultaneously, antioxidants scavenge ROS radicals, which are generated with unbalanced cellular homeostasis. Together, these mechanisms increased the accessibility of crop roots to water resources with maintained or enhanced root growth (Kang et al., 2022). The augmentation of root attributes, specifically, root length, root volume, and root area, holds the potential to significantly increase crop yield under drought conditions. In sorghum, it has been demonstrated that silicon treatment leads to an increase in polyamine accumulation and a decrease in ethylene production. This, in turn, results in delayed leaf aging and increased root growth relative to the shoot (Yin et al., 2014). Interestingly, such effects were not prominently observed in the appearance of the above-ground parts of plants. Visual effects, in the form of reduced leaf turgor, were noticeable primarily between the group of plants experiencing drought compared to those growing in optimal conditions. Based on these observations, it can be hypothesized that silicon supplementation of oilseed rape plants during drought may more actively stimulate the metabolism of their roots compared to their shoots. Moreover (Sheng et al., 2018) found that the deposition of Si in plants visibly changes the structure of their cell wall, causing NH4^+^ capture. This means that silicon ensures greater stability of the cell wall, leading to more optimized nutrient uptake, which is conducive to the strengthening, growth and development of supplemented plants. However, silicon-mediated root growth modification is not a common phenomenon for all plants and it remains unclear whether or not Si is directly involved in this process (Chen et al., 2018).

Under drought, as in the case of other, mainly abiotic, stresses, an important role is played by processes related to water management – both its intra- and intercellular as well as intra- and inter-tissue transport (Banerjee and Roychoudhury, 2020). As water is essential for photosynthesis, its optimized circulation is necessary for the stabilization of physiological processes in plants (Banerjee and Roychoudhury, 2016). These functions are largely handled by aquaporins – membrane proteins acting as transmembrane channels enabling the transport of, among others, water (Surbanovski and Grant, 2014). Under drought, the fastest responders to changes in water management are the PIP and TIP aquaporins (Afzal et al., 2016), which are involved in short-distance water transport in order to maintain cell turgor. In the leaves of oilseed rape plants supplemented with silicon under drought stress, it was found that there was a reduction in the accumulation of BnPIP2-1-7 aquaporin (Saja-Garbarz et al., 2022). In order to illustrate the entirety of the changes, this knowledge on the functioning of PIP1 and PIP2 proteins needed to be supplemented with analyses performed on the roots. This is particularly important due to the fact that, as previously observed in rice plant cells, reduced expression of aquaporin genes prevents the loss of metabolic energy in situations of severe stress and/or water loss from the root to the surrounding hypertonic environment (Sonah et al., 2017). Plants in which PIP genes were silenced had a reduced root hydraulic system (Martre et al., 2002). On the other hand, in many plant species an overexpression of *PIP* and *TIP* genes is observed under drought stress conditions (Alexandersson et al., 2010; Xu et al., 2014; Li et al., 2015; Sonah et al., 2017). A verification of these relationships in oilseed rape could significantly improve our understanding of silicon’s mechanism of action in this species. However, the analyses performed on oilseed rape roots did not yield results allowing to conclusively and unambiguously compare the levels of PIP1 and PIP2 accumulation in the roots of plants supplemented with Si and the control (**Supplementary Figure 2**). The protein content in the extract used for analyses was insufficient to apply the appropriate amount of protein for quantitative analysis. Consequently, the transcription of aquaporin genes in the roots was not analyzed in this study. However, this analysis is planned for further experiments. Drawing from our experience with biochemical and molecular analyses on the roots of *B. napus*, we can deduce that isolating a substantial amount of protein from these organs is challenging. Therefore, in future studies, it is imperative to collect a larger amount of plant material and employ concentration methods for protein extracts obtained from *B. napus* roots.

This study focused primarily on the changes in the accumulation of the tonoplast aquaporin – TIP. It was demonstrated for the first time that in oilseed rape roots under drought the accumulation of BnTIP1;1 decreases compared to that observed in plants growing in well-watered conditions, and that this effect is enhanced in plants supplemented with silicon (**Figure 2**). A conclusion can be drawn that the limited accumulation of water channels in tonoplast membranes caused by silicon increases water retention in cells by limiting its outflow and thus better protects plants against drought stress. This interpretation is confirmed by the previously discussed results showing better water relations under drought in plants supplemented with silicon compared to the control, evaluated on the basis of RWC (**Table 1**). Additionally, the good metabolic condition of plants treated with Si under drought is also evidenced by the increased growth of their roots compared to plants not supplemented with silicon (**Supplementary Figure 1**), which was also demonstrated by Chen et al. (2018). However, as the plant responses in both the accumulation of proteins and the expression of aquaporin genes are complex and wide-ranging, further experimental studies are required in order to draw more comprehensive conclusions about the mechanisms regulating these processes.

Drought causes disturbances in the plant’s water balance and therefore also in the metabolic processes, which results in an increase in the production of ROS and, consequently, in oxidative stress. The metabolic response of shoots to drought contrasts with that of roots. It has been demonstrated that shoots undergo metabolic deactivation under drought to reduce water and nutrient consumption, while roots are metabolically activated to enhance water and nutrient uptake. These processes collectively act to buffer the effects of drought (Gargallo-Garriga et al., 2014). In the current study, a similar effect was observed concerning changes in catalase (CAT) activity during drought stress in plants supplemented with silicon. However, the changes in superoxide dismutase (SOD) activity, although observed, were not found to be statistically significant. The antioxidant mechanisms appear to provide crucial protection against oxidative damage in cell membranes and organelles in cells of plants growing under unfavorable conditions. Plants are equipped with a complex and highly efficient antioxidant defense system able to respond and adapt to drought stress, consisting of non-enzymatic and enzymatic protective mechanisms which effectively scavenge ROS and prevent the damaging effects of free radicals. It has been demonstrated that silicon plays an important role in inducing the activity of the antioxidant system in the plant’s defense response to environmental stress (Gunes et al., 2008; Saja-Garbarz et al., 2022). Si supplementation of plants subjected to unfavorable conditions intensifies the antioxidant activity of cells, leading to detoxification of excess ROS and maintenance of homeostasis necessary for the proper functioning of the living cell (Wu et al., 2017; Saja-Garbarz et al., 2022). In conditions of water scarcity, silicon affects the mechanisms of antioxidant regulation depending on the species and the plant development stage (Gong et al., 2008). The high activity of many SOD isoforms identified in oilseed rape roots was not additionally stimulated by the presence of silicon (**Figure 3**), in contrast to changes observed in leaves of oilseed rape plants subjected to drought (Saja-Garbarz et al., 2022) and oilseed rape seedlings under salinity stress (Hasanuzzaman et al., 2018). This could be explained on the basis of results indicating translocation and higher accumulation of silicon in leaves of supplemented plants than in roots, where it can elevate the SOD genes expression level. It was previously documented in wheat leaves that Si plays a pivotal role in the improvement of transcriptional activity of several antioxidant enzymes genes among them *TaSOD* in response to drought (Ma et al., 2016). On the other hand, both in well-watered conditions (a tendency in the case of silicon and a significant difference in the case of silicon complex) and under drought, supplementation of oilseed rape with Si was observed to stimulate the enzymatic activity of CAT in the roots (**Figure 4**). Previously, Dai et al. (2020) also found an increase in CAT activity under drought in two genotypes of oilseed rape treated with melatonin as a factor ameliorating oxidative stress. The increase in CAT activity correlates with intensive plant root growth indicating their high metabolic activity under drought. Moreover, it is also a frequently reported phenomenon after silicon supplementation of plants subjected to various stress factors (Abbas et al., 2015; Hasanuzzaman et al., 2018; Ahmad et al., 2019). Additionally, it was shown that stomatal conductance correlated significantly and negatively with the activity of CAT in roots (**Supplementary Table SM6**), which could mean that partial stomata closure (decrease in stomatal conductance) increases CAT activity. It might be connected with higher activity of roots metabolism due to their intensive growth and their higher water uptake ability, which compensates for lower suction force generated by stomata transpiration. This effect did not depend on silicon supplementation. The activity of low molecular weight antioxidants in the roots increased after Si supplementation in well-watered conditions (the most in silicon complex plants), but this effect was not observed under drought (**Figure 5**). This might be associated with high consumption of the products of primary metabolism, which serve as a source for secondary metabolites (e.g. phenols and polyphenols) being a part of the antioxidant pool measured in the analysis of total antioxidant activity.

Our results regarding silicon supplementation and antioxidant responses in oilseed rape plants align with existing research on other plants, particularly in terms of the potential of silicon supplementation to mitigate the effects of drought stress. However, in some instances, ambiguity in the obtained results and discrepancies between plants supplemented with orthosilicic acid and those supplemented with *Optysil* may arise from the potential high sensitivity of rapeseed to the presence of additional ions besides silicon. The ameliorating effect of the combined application of Si with other ions, e.g., SO_4_, has been investigated in other plant species (Nisar et al., 2023), and it has been demonstrated to alleviate the adverse impact of drought stress on the growth and development of sunflowers. Overall, the application of Si and SO_4_ has shown significantly higher potential for increasing sunflower productivity and influencing the increase of the plant’s antioxidant activity under drought conditions compared to sole Si supplementation. Nonetheless, confirming this hypothesis requires further research.

## 5 Conclusions

In conclusion, the study reveals that silicon plays a stimulatory role in enhancing the growth of the root system, thereby increasing their absorptive surface. It improves the water balance in oilseed rape plants under drought stress by promoting water uptake through the roots while concurrently enhancing water retention in the cells. These processes are intricately regulated by tonoplast aquaporins, a novel finding highlighted in this study. The observed reduction in the accumulation of TIP aquaporins during drought, particularly after silicon supplementation, underscores the crucial role of silicon in potentially regulating water transport in the plant. This is noteworthy, even considering that oilseed rape is not a plant known for efficient silicon accumulation. The diminished accumulation of TIP aquaporins suggests that the plant actively limits water flow during drought, indicating an active protective mechanism to reduce water losses at the plant-soil interface. Additionally, the study demonstrates that silicon stimulates catalase activity in plants grown under both well-watered and drought conditions. However, the most significant increase in catalase activity is found under stress conditions, emphasizing catalase’s role in alleviating oxidative stress. Furthermore, it is shown that the content of low molecular weight antioxidants is not influenced by silicon treatment in drought conditions. Still, their content is significantly increased by silicon in well-watered conditions. This finding affirms previous research, indicating that aside from ameliorating oxidative stress, silicon also influences secondary metabolism, such as phenol content, in non-stressed plant tissues.

## Data availability statement

The raw data supporting the conclusions of this article will be made available by the authors, without undue reservation.

## Author contributions

DS: Conceptualization, Data curation, Formal analysis, Funding acquisition, Investigation, Methodology, Project administration, Resources, Software, Supervision, Validation, Visualization, Writing – original draft, Writing – review & editing. ML: Investigation, Supervision, Validation, Visualization, Writing – original draft, Writing – review & editing. FJ: Investigation, Methodology, Supervision, Validation, Visualization, Writing – review & editing.
