# Supplementary material for: Silicon improves root functioning and water management as well as alleviates oxidative stress in oilseed rape under drought conditions
Source: Front Plant Sci. 2024 Feb 21;15:1359747. doi: 10.3389/fpls.2024.1359747 (PMC10915341; doi:10.3389/fpls.2024.1359747)
Supplement: Supplementary file 1 [file DataSheet_1.docx]

**Supplementary Material**

**Figures**


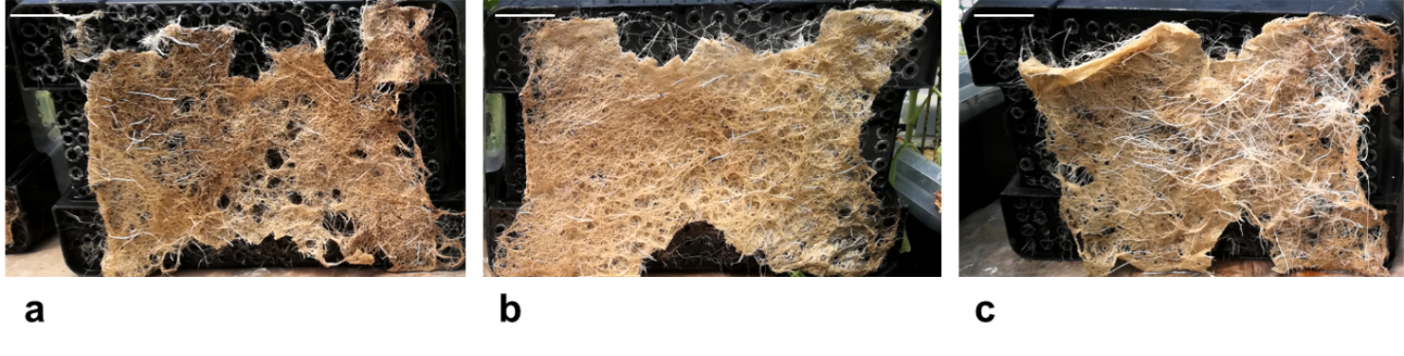


Figure SM1. Roots of 54-day-old oilseed rape plants after growing under drought for 10 days. Prior to the drought treatment, the plants grew for 44 days in well-watered conditions and were watered three times with water (control) (a), silicon (b), and silicon complex solution (c).


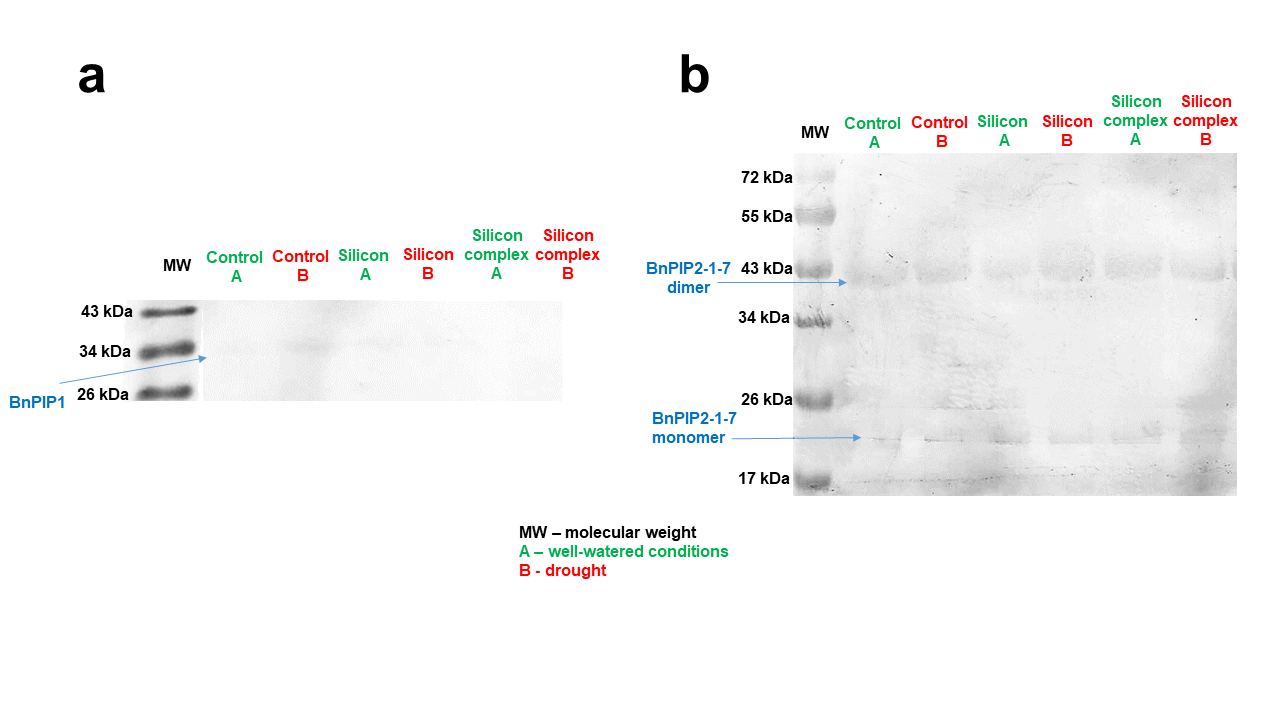


Figure SM 2. The impact of drought on the accumulation of BnPIP1 (a) and BnPIP2-1-7 (b) proteins in the roots of control and silicon-treated oilseed rape plants. The visualized band intensity corresponds with the level of protein identified with anti-PIP1 (a) and anti-PIP2-1-7 (b) antibody. MW – molecular weight standard (Thermo Scientific PageRuler Prestained Protein Ladder), 10 µg of total proteins were applied on each lane.


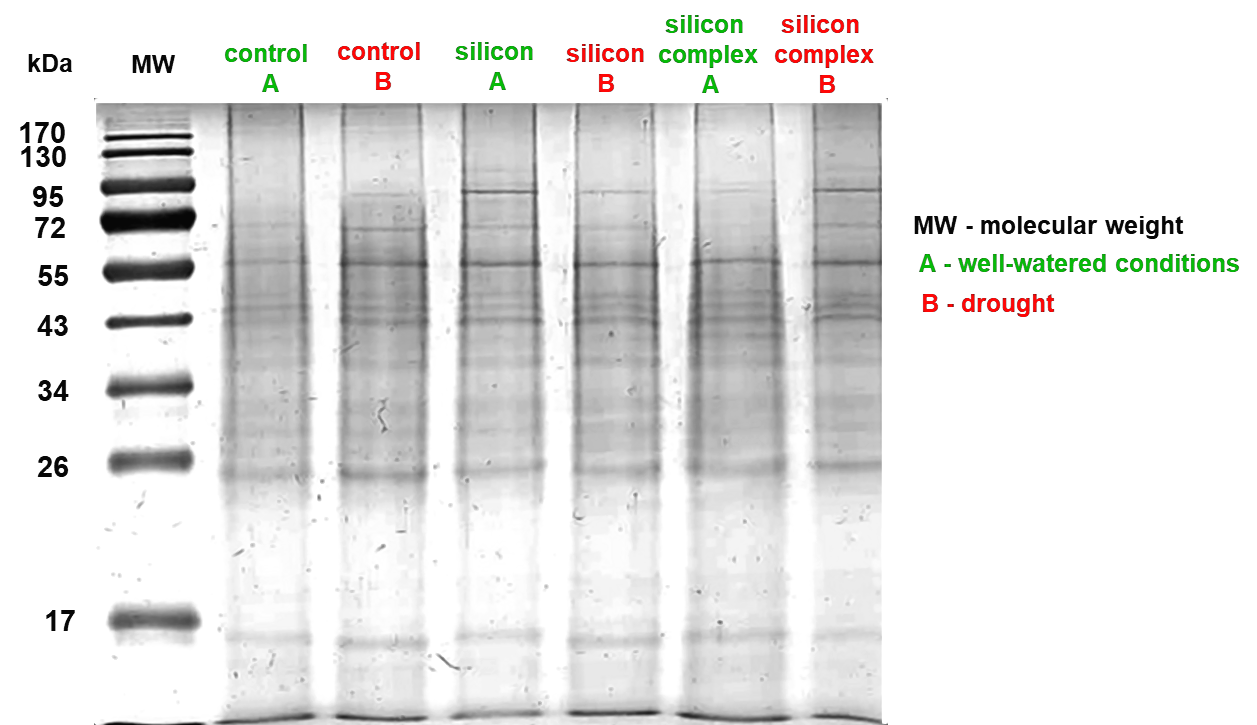


Figure SM3. Gel stained with Coomassie Brilliant Blue solution after electrophoretic separation of proteins isolated from the studied plant material. Equal amount of protein fraction isolated from roots of oilseed rape plants growing under well-watered and drought conditions, watered with water (control), silicon or silicon complex. 10 µg was loaded on the gel. MW – molecular weight standard (Thermo Scientific PageRuler Prestained Protein Ladder).

Tables

Table SM1. Silicon content and selected parameters of water management (silicon content; leaf relative water content, leaf osmotic potential, stomatal conductance, root osmotic potential, root abscisic acid level) in oilseed rape plants. The impact of silicon supplementation was examined in roots and leaves of plants growing under well-watered and drought conditions. Control – plants not supplemented with silicon. The results of two-factor analysis of variance (ANOVA) are presented. The sources of variance of the measured parameters were as follows: two growth conditions, three treatments, and their interactions. * significant at p ≤ 0.05; ns, not significant.

| **Source of variance** | **Root silicon content (mg/kg)** | **RWC (%)** | **Leaf osmotic potential (MPa)** | **Stomatal conductance (mmol m^-2^ s^-1^)** | **Root osmotic potential (MPa)** | **Root abscisic acid level (nmol g^-1^DW)** |
| --- | --- | --- | --- | --- | --- | --- |
| **Growth conditions** | ns | * | * | * | * | ns |
| **Treatments** | * | * | * | ns | * | ns |
| **Growth conditions*Treatments** | * | * | * | * | * | * |

Table SM2. The impact of drought on the accumulation of BnTIP1;1 protein in roots of control and silicon-supplemented plants of oilseed rape. The results of two-factor analysis of variance (ANOVA). The sources of variance of the measured parameters were as follows: two growth conditions, three treatments, and their interactions. * significant at p ≤ 0.05; ns, not significant.

| **Source of variance** | **BnTIP1;1 accumulation (A.U.)** |
| --- | --- |
| Growth conditions | * |
| Treatments | * |
| Growth conditions*Treatments | * |

Table SM3. The impact of silicon supplementation on SOD activity in protein fraction isolated from the roots of oilseed rape plants growing in well-watered and drought conditions. Control – plants not supplemented with silicon. The results of two-factor analysis of variance (ANOVA). The sources of variance of the measured parameters were as follows: two growth conditions, three treatments, and their interactions. * significant at p ≤ 0.05; ns, not significant.

| **Source of variance** | **SOD activity (A.U.)** |
| --- | --- |
| Growth conditions | ns |
| Treatments | ns |
| Growth conditions*Treatments | ns |

Table SM4. The impact of silicon supplementation on CAT activity in protein fraction isolated from roots of oilseed rape plants growing in well-watered and drought conditions. Control – plants not supplemented with silicon. The results of two-factor analysis of variance (ANOVA). The sources of variance of the measured parameters were as follows: two growth conditions, three treatments, and their interactions. * significant at p ≤ 0.05; ns, not significant.

| **Source of variance** | **CAT activity (A.U.)** |
| --- | --- |
| Growth conditions | * |
| Treatments | * |
| Growth conditions*Treatments | * |

Table SM5. The impact of silicon supplementation on total non-enzymatic antioxidant activity in roots of oilseed rape plants growing in well-watered and drought conditions. Control – plants not supplemented with silicon. The results of two-factor analysis of variance (ANOVA). The sources of variance of the measured parameters were as follows: two growth conditions, three treatments, and their interactions. * significant at p ≤ 0.05; ns, not significant.

| **Source of variance** | **Non-enzymatic antioxidants (µM of Trolox equivalents/g DW)** |
| --- | --- |
| Growth conditions | * |
| Treatments | * |
| Growth conditions*Treatments | * |

Table SM6. Pearson’s correlation coefficients (r) among all traits assessed on the basis of their the means values. Analysis was performed on roots and leaves of plants growing for 54 days under well-watered conditions and drought – effect of silicon. Control – plants untreated with silicon. * , ** significant at p ≤ 0.05, p ≤ 0.01, respectively; ns, not significant. Green indicates a positive correlation and yellow indicates a negative correlation.

| PARAMETERS | Root silicon content (mg/kg) | RWC (%) | Leaf osmotic potential Ψo (MPa) | Stomatal conductance (mmol m^-2^ s^-1^) | Root osmotic potential Ψo (MPa) | Root abscisic acid level (nmol g^-1^_DW_) | BnTIP1;1 accumulation (A.U.) | SOD activity (A.U.) | CAT activity (A.U.) | Non-enzymatic antioxidants  (µM of Trolox equivalents/g DW) |
| --- | --- | --- | --- | --- | --- | --- | --- | --- | --- | --- |
| Root silicon content (mg/kg) | X |  |  |  |  |  |  |  |  |  |
| RWC (%) | ns | X |  |  |  |  |  |  |  |  |
| Leaf osmotic potential Ψo (MPa) | ns | * | X |  |  |  |  |  |  |  |
| Stomatal conductance  (mmol m^-2^ s^-1^) | ns | ns | ns | X |  |  |  |  |  |  |
| Root osmotic potential Ψo (MPa) | ns | ns | * | ns | X |  |  |  |  |  |
| Root abscisic acid level  (nmol g^-1^_DW_) | ns | ns | ns | ns | ns | X |  |  |  |  |
| BnTIP1;1 (A.U.) | ns | ns | * | ns | ns | ns | X |  |  |  |
| SOD (A.U.) | ns | ns | ns | * | ns | ns | ns | X |  |  |
| CAT (A.U.) | ns | ns | ** | * | ns | ns | * | ns | X |  |
| Non-enzymatic antioxidants  (µM of Trolox equivalents/g DW) | ns | ns | ns | ns | ns | ns | ns | ns | ns | X |
